# The First-Reported Case of Drug-Induced Hemolytic Anemia by Piperacillin-Tazobactam in a Premature Neonate: A Case Report and Literature Review

**DOI:** 10.7759/cureus.35915

**Published:** 2023-03-08

**Authors:** Hamza Salim, Basel Musmar, Fajr M A Sarhan, Narmeen Giacaman, Shatha Abu Omar

**Affiliations:** 1 Department of Medicine, An-Najah National University, Nablus, PSE; 2 Department of Internal Medicine, Al-Quds University, Abu-Dis, PSE; 3 Department of Pediatrics, Thabit Thabit Hospital, Tulkarem, PSE

**Keywords:** hemolytic disease of the newborn, case report, penicillin allergy, warm autoimmune hemolytic anemia, immune hemolytic anemia, premature infants, drug-induced immune hemolytic anemia, drug-induced hemolytic anemia, piperacillin-tazobactam

## Abstract

Drugs can have a wide array of effects on hematological cells, including red blood cells (RBCs), white blood cells (WBCs), and platelets. Drug-induced hemolytic anemia (DIHA) can be explained by three different pathophysiological mechanisms. We present a case of a premature neonate born at 34 weeks gestation who was admitted to the neonatal intensive care unit (NICU). He developed respiratory difficulty with mottled skin and was suspected to have bacterial sepsis due to necrotizing enterocolitis (NEC). The patient was eventually started on a broad-spectrum antibiotic, piperacillin-tazobactam. On day eight, the patient started developing jaundice and his hemoglobin level dropped from 12.1 to 8.2 mg/dL. His direct antiglobulin test (DAT) was strongly positive. The patient was suspected to have DIHA. Piperacillin-tazobactam is a commonly used antibiotic for neonatal sepsis, but its potential to cause DIHA in neonates is not well-established. Our case highlights the importance of considering piperacillin-tazobactam as an unrecognized contributor to neonatal jaundice and a potential cause of DIHA in neonates. Further research is needed to explore the extent of its involvement in this condition. Physicians should be cautious when administering this drug to neonates and be aware of the possibility of hemolysis and jaundice.

## Introduction

Neonatal jaundice is a common condition that affects up to 60% of term and 80% of preterm newborns worldwide. The majority of cases are benign and self-limited, but in some instances, it can be indicative of an underlying pathology [1]. Piperacillin-tazobactam is a widely used antibiotic in neonatal intensive care units (NICUs) and has been implicated in causing drug-induced hemolytic anemia (DIHA) in adults. However, its role in neonatal DIHA is not well-established.

In this case report, we present a case of a neonate who developed jaundice and hemolytic anemia after receiving piperacillin-tazobactam treatment for suspected sepsis. We highlight the potential for piperacillin-tazobactam to induce DIHA in neonates and the need for clinicians to be aware of this potential adverse effect. We also discuss the subclinical possibility of piperacillin-tazobactam-induced hemolysis and the need for further research to better understand its incidence and clinical significance. To the best of our knowledge, this is the first case report of isolated neonatal hemolytic anemia caused by piperacillin-tazobactam. 

## Case presentation

A single, live, premature caucasian male was born at 34 weeks of gestation via emergency cesarean section to a gravida 2 para 1 mother for preterm labor with a previous history of cesarean section. His APGAR (appearance, pulse, grimace, activity, and respiration) scores were 8 and 9 at 1 and 5 minutes, respectively. His birth weight was 1.6 kg. After birth, the baby was admitted to the neonatal intensive care unit (NICU) and then referred to our hospital to continue the NICU care at the age of four days.

On arrival at our hospital, he showed signs of mild respiratory distress. His saturation was 98% on O2 nasal cannula. Workup was carried out on arrival, which showed normal electrolytes, normal blood sugar, a hemoglobin level of 14.6 mg/dL, a mean corpuscular hemoglobin concentration (MCHC) of 32.7 g/dl, with a negative direct antiglobulin test (DAT), and mild indirect hyperbilirubinemia. Supportive care consisting of IV fluids was initiated in addition to the administration of ampicillin and cefotaxime. The patient is a member of a family of four individuals, with healthy parents and one healthy older sibling. There was no reported family history of hematologic or autoimmune conditions. There was no consanguinity between the parents, and no significant psychosocial history was identified.

On the second day of hospitalization, he developed apnea, his O2 saturation was 81%, his temperature was 37.6 °C, his blood pressure was 73/29, and he had tachycardia. The patient developed cyanosis, abdominal distention, and mottled skin. A septic workup was conducted. The blood culture result was negative and the urinalysis was normal; an abdominal X-ray showed distention and no other signs of necrotizing enterocolitis were evident. Stool occult blood was negative and his chest X-ray was clear. Given the concern of “clinical sepsis”, cefotaxime was changed to piperacillin-tazobactam. Amikacin was added, as the NICU had extended-spectrum beta-lactamase bacterial colonization. Ampicillin was continued.

**Figure 1:**
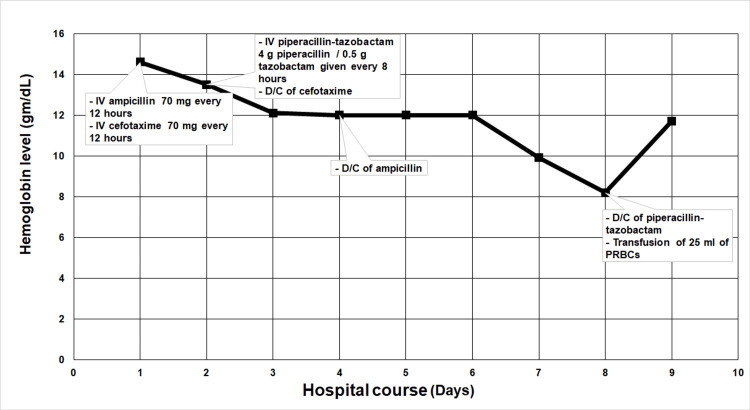
Changes in hemoglobin levels throughout the course of hospitalization and their relationship with the administered antibiotics

On day eight of hospitalization (12 days old), the patient was noticed to develop signs of jaundice, pallor, and darkening of his urine, but his vital signs were stable; at that time, he had continued to receive IV piperacillin-tazobactam. A jaundice workup was carried out he was found to have indirect hyperbilirubinemia his total bilirubin was elevated to 11.7 (last measure was 7.7 mg/dl), His hemoglobin was 8.2 mg/dL (declined from a baseline of 12.1 mg/dL) (Figure 1), his urinalysis showed trace hemoglobinuria with negative RBCs in the urine. Hemolysis was suspected to be the cause of jaundice in this preterm neonate; the direct antiglobulin test (DAT) was strongly positive for immunoglobulin G (IgG). A blood film showed spherocytosis with no evidence of schistocytes, sickle cells, Heinz bodies, or bite cells, his lactate dehydrogenase (LDH) was 1146 U/L, which is elevated, the patient’s blood group is O+ and the same as his mother (O+). His white blood cells (WBCs), platelets, serum electrolytes, random blood sugar, creatinine, liver function tests, thyroid-stimulating hormone (TSH), and coagulation studies were within normal ranges.

Piperacillin-tazobactam was suspected to be the causative agent of hemolysis. Thereafter, 25 ml of compatible packed red blood cells (PRBCs) were transfused, piperacillin-tazobactam administration was discontinued, and phototherapy was started. The patient’s hemolysis gradually subsided and his hemoglobin level stabilized.

After a week, the patient was brought to the clinic healthy; vital signs and physical examination were normal, his jaundice was resolved, his complete blood count was normal, his hemoglobin level was 12 mg/dL, and he did not require any further treatment. Four months later, the patient remained in good health, with a hemoglobin level of 11.2 mg/dL and no significant findings.

## Discussion

Drugs can have a wide range of effects on WBCs, red blood cells (RBCs), and platelets. The effect on RBCs can range from asymptomatic hemolytic anemia to life-threatening DIHA and death [2]. DIHA is a rare condition with an estimated incidence of one to four cases per million per year. A study reported that there have been over 130 drugs associated with DIHA such as cephalosporins, penicillins, and so on [3]. Although second and third-generation cephalosporins are among the most common causes of DIHA, piperacillin-tazobactam is also a culprit [4]. The mechanism behind DIHA can be traced to three different etiologies: a drug-dependent mechanism that creates a hapten, autoantibody mediation, or immune-complex mediation. Penicillins typically have a drug-dependent mechanism leading to complement fixation and intravascular lysis [5].

A review of prior studies and reports showed that piperacillin-tazobactam-induced hemolytic anemia is more common in patients with cystic fibrosis, which may be attributed to their repeated exposure to recurrent infections [6,7]. A recent study has reported an instance of piperacillin-tazobactam-induced pancytopenia in a neonate. The underlying cause was identified as maternal human leukocyte antigen (HLA) antibodies present in the mother diagnosed with Sjogren's syndrome [8]. To our knowledge, this is the first reported case of isolated neonatal hemolytic anemia resulting from piperacillin-tazobactam.

There are two potential mechanisms that may contribute to the development of DIHA in piperacillin-tazobactam combination therapy: immunologic and non-immunologic. The immunologic mechanism is primarily attributed to piperacillin, which coats RBCs and results in the production of anti-piperacillin antibodies that can be of the IgG and/or IgM type. These antibodies then activate the complement system and cause hemolysis. Interestingly, studies have shown that individuals with Rh(e)+ blood group (as in our case) are more susceptible to the development of DIHA due to the preference of piperacillin antibodies for Rh(e)+ RBCs [9]. Moreover, some studies showed that piperacillin has the potential to cause both intra and extravascular hemolysis [10].

On the other hand, the adverse reaction to piperacillin-tazobactam may be linked to non-immunologic protein adsorption (NIPA) induced by tazobactam, the beta-lactamase inhibitor [11]. Tazobactam is known to cause NIPA leading to hemolytic anemia. Studies have demonstrated that beta-lactamase inhibitors such as tazobactam, sulbactam, and clavulanate can cause the adsorption of proteins onto RBC membranes, which can lead to positive DAT. The non-specific uptake of beta-lactamase inhibitor protein onto RBCs in vivo may sometimes lead to decreased RBCs survival which subsequently leads to hemolysis, which can be more severe in premature neonates who already have a decreased life span of RBCs [12]. This hypothesis is supported by studies with sulbactam and clavulanate-treated RBCs [13].

Neonates' immune system differs from adults in whom neonatal B-cells are immature and have limited antibody production due to a partially developed surface immunoglobulin repertoire [14]. Given this, it is unlikely that the response was due to neonatal B-cell antibody production against piperacillin. The more probable cause is non-immunologic protein adsorption (NIPA) due to transplacental maternal antibody reaction with RBCs caused by tazobactam adsorption. In a previous study, a comparable unfavorable occurrence was reported with sulbactam, which led to the death of a neonate as a result of hemolysis caused by non-immunologic protein adsorption (NIPA) [15]. Cephalosporins' similar mechanism of DIHA is noteworthy, but it is unlikely to have contributed to this hemolysis, as it was discontinued seven days prior [9].

When evaluating a neonate with jaundice, it is important to consider hemolysis as a possible cause [16]. Hemolysis in neonates can be due to intrinsic defects in RBCs or external factors such as infections, medications, or physical disruption of RBCs in blood vessels [12]. In this particular case, ABO and Rh incompatibility were ruled out as potential causes due to both the neonate and the mother having the same blood group (O+) and no history of Rh incompatibility, as well as the presentation of symptoms at 12 days of age. Spherocytosis on the blood film and a positive DAT indicated an immune reaction, rather than hereditary spherocytosis. Other intrinsic defects in RBCs, hemoglobinopathies, and warm autoimmune hemolytic anemia (AIHA) were deemed unlikely based on the acute onset of symptoms, blood film findings, and resolution of anemia after discontinuation of the medication [17].

Early recognition and management of DIHA are essential to lower its associated morbidity and mortality. DIHA is commonly observed to occur within seven to 13 days of piperacillin-tazobactam therapy [10]. The clinical presentation of DIHA may vary, and patients may present with symptoms such as weakness, palpitations, pallor, and jaundice, among others. In our case, the neonate presented with jaundice, pallor, and stable vital signs. The complications of DIHA can range from disseminated intravascular coagulation and hemoglobinuria-induced acute renal failure, to shock. Therefore, prompt recognition and management of DIHA are essential to prevent its associated morbidity and mortality [18].

Diagnostic methods for hemolysis are more challenging to implement or yield less definitive results in preterm infants as compared to term infants. In the evaluation of neonatal jaundice suspected to be caused by hemolysis, laboratory tests are necessary to confirm the underlying etiology. Although urinalysis is typically used to detect hemoglobinuria, the presence of RBCs can invalidate the test, which is a common occurrence in neonates. Additionally, serum haptoglobin levels may be low or absent in preterm neonates. DAT can be a useful diagnostic tool, as well as an elevated absolute reticulocyte count. Abnormal RBC morphology on a stained blood film can also support a diagnosis of hemolysis [12]. If the more typical causes of hemolysis have been eliminated and there is a temporal correlation between drug initiation and the onset of hemolysis, an antibody elution test and drug antibody evaluations may be necessary [19].

Management depends on four important pillars: discontinuation of the causative drug; providing appropriate therapy, which consists of corticosteroids and intravenous immunoglobulin; blood transfusions; and management of jaundice as appropriate. In our patient, the discontinuation of piperacillin-tazobactam and transfusion of 25 ml of PRBCs in addition to phototherapy were enough to subside the hemolysis. Lactate dehydrogenase levels can be used to detect the severity of hemolysis and check the patient’s response to therapy [2,16,18].

## Conclusions

Our case study highlights the potential risk of piperacillin-tazobactam-induced DIHA in neonates, which can range from asymptomatic to life-threatening hemolysis, disseminated intravascular coagulopathy, and/or respiratory or renal failure. Although neonatal jaundice is a common condition with multiple contributing factors, we suspect that piperacillin-tazobactam-induced hemolysis can be a subclinical contributor to neonatal jaundice that is often missed. Further research is necessary to determine the extent of piperacillin-tazobactam's role in neonatal hemolysis and subsequent jaundice. We recommend that physicians should be cautious when administering this drug to neonates with jaundice or those at risk of developing it.
